# Probing the Scope and Mechanisms of Calcitriol Actions Using Genetically Modified Mouse Models

**DOI:** 10.1002/jbm4.10434

**Published:** 2020-12-05

**Authors:** Dengshun Miao, David Goltzman

**Affiliations:** ^1^ The Research Center for Aging Affiliated Friendship Plastic Surgery Hospital of Nanjing Medical University Nanjing China; ^2^ Department of Medicine McGill University Health Centre and McGill University Montreal QC Canada

**Keywords:** DENTAL BIOLOGY, GENETIC ANIMAL MODELS, OSTEOMALACIA AND RICKETS, OSTEOPOROSIS, PTH/VIT D/FGF23

## Abstract

Genetically modified mice have provided novel insights into the mechanisms of activation and inactivation of vitamin D, and in the process have provided phenocopies of acquired human disease such as rickets and osteomalacia and inherited diseases such as pseudovitamin D deficiency rickets, hereditary vitamin D resistant rickets, and idiopathic infantile hypercalcemia. Both global and tissue‐specific deletion studies leading to decreases of the active form of vitamin D, calcitriol [1,25(OH)_2_D], and/or of the vitamin D receptor (VDR), have demonstrated the primary role of calcitriol and VDR in bone, cartilage and tooth development and in the regulation of mineral metabolism and of parathyroid hormone (PTH) and FGF23, which modulate calcium and phosphate fluxes. They have also, however, extended the spectrum of actions of calcitriol and the VDR to include, among others: modulation, jointly and independently, of skin metabolism; joint regulation of adipose tissue metabolism; cardiovascular function; and immune function. Genetic studies in older mice have also shed light on the molecular mechanisms underlying the important role of the calcitriol/VDR pathway in diseases of aging such as osteoporosis and cancer. In the course of these studies in diverse tissues, important upstream and downstream, often tissue‐selective, pathways have been illuminated, and intracrine, as well as endocrine actions have been described. Human studies to date have focused on acquired or genetic deficiencies of the prohormone vitamin D or the (generally inactive) precursor metabolite 25‐hyrodxyvitamin D, but have yet to probe the pleiotropic aspects of deficiency of the active form of vitamin D, calcitriol, in human disease. © 2020 American Society for Bone and Mineral Research © 2020 The Authors. *JBMR Plus* published by Wiley Periodicals LLC on behalf of American Society for Bone and Mineral Research.

## Introduction

Genetically modified mouse models, including both global deletion, as well as conditional deletion of relevant genes, have reinforced and expanded the major action of the calcitriol, ie, 1,25‐dihydroxyvitamin D [1,25(OH)_2_D]/vitamin D receptor (VDR) pathway in modulating mineral and skeletal homeostasis, and have permitted more controlled and extensive examination of the phenotypes and underlying mechanisms than are possible by examination of the corresponding human disorders alone. They have also disclosed potentially critical extraskeletal actions.

## Insights into Vitamin D Metabolism

**Fig 1:**
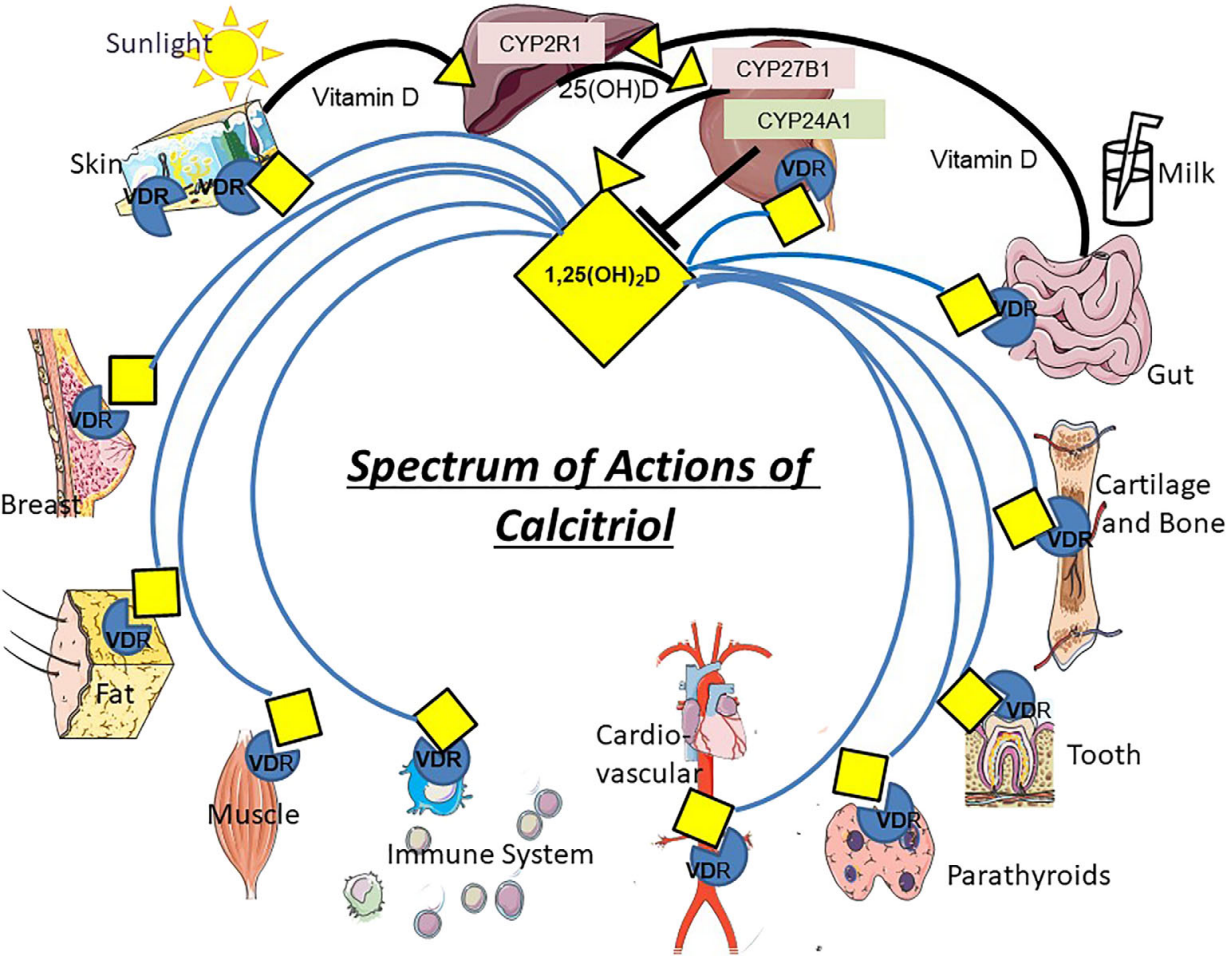
Model of calcitriol generation and action. Vitamin D may be obtained by sunlight exposure or absorption from the gut and metabolized sequentially, by liver CYP2R1 and kidney (or extrarenal) CYP 27B1, to calcitriol (1,25(OH)_2_D). 1,25(OH)_2_D may be degraded by CYP24A1. 1,25(OH)_2_D may act via the VDR in the multiple tissues noted, although the best documented are tissues of the skeletal system and gut. The VDR may act independently in skin.

Vitamin D undergoes serial metabolic transformations to an active entity, 1,25(OH)_2_D. Thus, vitamin D, produced in skin as cholecalciferol (vitamin D3), or absorbed from the gut as vitamin D_3_ or as ergocalciferol (vitamin D_2_), is transported in the circulation, bound to a vitamin D binding protein (DBP). In the liver, vitamin D can be 25‐hydroxylated, mainly by the enzyme CYP2R1, to produce a metabolite, 25‐hydroxyvitamin D [25(OH)D], which is generally inactive at normal circulating concentrations, but which may be active at very high concentrations. *CYP2R1* loss‐of‐function mutations in humans result in vitamin D–deficiency rickets,^(^ ^1^ ^)^ and mice with *Cyp2r1* deletion show an approximately 50% reduction in serum 25(OH)D concentrations compared with wild‐type mice.^(^ ^2^ ^)^ 25(OH)D, the most abundant circulating form of vitamin D, can then be converted to 1,25(OH)_2_D by the action of CYP27B1, the 25‐hydroxyvitamin D‐1α‐hydroxylase [1α(OH)ase] (Fig. 1).

Initial mouse models examining global deletion of *Cyp27b1*, [1α(OH)ase^−/−^ mice], produced a phenocopy of the congenital human disease vitamin D–dependent rickets type 1A (VDDRIA), also known as pseudovitamin D deficiency rickets (PDDR).^(^ ^3^, ^4^ ^)^ The major source of circulating 1,25(OH)_2_D is the renal 1α(OH)ase enzyme, which is tightly upregulated by parathyroid hormone (PTH) and hypophosphatemia, and downregulated by the phosphaturic hormone fibroblast growth factor 23 (FGF23), by hypercalcemia, and by 1,25(OH)_2_D itself. Although circulating concentrations of 1,25(OH)_2_D were undetectable in the homozygous *Cyp27b1* null mice, serum 25(OH)D concentrations were elevated, likely reflecting absence of metabolizing enzymes, and, serum calcium and phosphate concentrations were reduced, consequent to decreased intestinal absorption, and secondary hyperparathyroidism with phosphaturia.^(^ ^3^ ^)^

The active form, 1,25(OH)_2_D, as well as 25(OH)D, can be metabolized, largely by CYP24A1, a 24‐hydroxylase [24(OH)ase], via a series of hydroxylation/oxidation reactions at carbons 24 and 23. Although the metabolite 1,24,25(OH)_3_D, which retains biological activity, may initially be generated, inactive calcitroic acid is ultimately produced. Indeed deletion of *Cyp24a1* in mice demonstrated the critical role of this enzyme in eliminating 1,25(OH)_2_D^(^ ^5^ ^)^ in that those mice that survived postweaning (~50%) had increased circulating 1,25(OH)_2_D levels. CYP24A1 can be upregulated by both 1,25(OH)_2_D and FGF23.

1,25(OH)_2_D as a steroidal hormone acts mainly as a ligand of the widely distributed nuclear vitamin D receptor (VDR). However, membrane‐localized VDR has also been reported to exhibit nongenomic actions.^(^ ^6^ ^)^ The genomic mechanism of 1,25(OH)_2_D action involves binding of 1,25(OH)_2_D to a VDR/retinoic X receptor (VDR/RXR) heterodimeric complex, which then binds to specific DNA sequences (Vitamin D response elements [VDREs]). A number of VDR co‐regulatory proteins have been identified, and epigenetic changes may also occur in histones that regulate access to transcriptional target genes. The actions of 1,25(OH)_2_D via VDR involve stimulation or suppression of gene activity, often quite distant from the transcription start site.^(^ ^7^ ^)^ Global deletion of the *Vdr* (Vdr^−/−^ mice) produced a phenocopy of the congenital human disease vitamin D dependent rickets type 2A (VDDR2A), also known as hereditary vitamin D resistant rickets (HVDRR).^(^ ^8^ ^)^

In global *Vdr* null mice, expression of renal 1α(OH)ase was elevated and 24(OH)ase was suppressed due to loss of 1,25(OH)_2_D action, resulting in high circulating 1,25(OH)_2_D concentrations. In the compound mutants, 1α(OH)ase^−/−^Vdr^−/−^, a “rescue” diet containing high calcium, high phosphorus, and 20% lactose appeared to increase calcium transport independent of the 1,25(OH)_2_D/Vdr system and normalized serum calcium^(^ ^9^ ^)^ Thus, the rescue diet may increase transport of calcium via genes not regulated by 1,25(OH)_2_D.^(^ ^10^ ^)^ Serum phosphate was also normalized as elevated PTH levels normalized. Elimination of hypocalcemia and hypophosphatemia alone, using a rescue diet, also normalized renal 24(OH)ase in 1α(OH)ase^−/−^ mice and normalized both renal 24(OH)ase as well as 1α(OH)ase levels in Vdr^−/−^ mice. This, therefore, demonstrated a calcium regulatory effect on gene expression of these enzymes independent of the 1,25(OH)_2_D/VDR system.

Cyp24a1^−/−^ mice exhibited increased 1,25(OH)_2_D levels, hypercalcemia, hypercalciuria, and an intramembranous bone lesion that healed when a double *Cyp24a1/Vdr* null mouse was generated, indicating that the elevated 1,25(OH)_2_D, acting through Vdr, was responsible for the bone defect.^(^ ^5^ ^)^ These observations in Cyp24a1^−/−^ mice preceded observations in humans demonstrating that inactivating mutations in *CYP24A1* are a cause of idiopathic infantile hypercalcemia,^(^ ^11^ ^)^ as well as a syndrome of intermittent hypercalcemia, hypercalciuria, nephrocalcinosis, and kidney stones, in some adults.^(^ ^12^ ^)^

## Intestinal Absorption of Calcium

Hypocalcemia occurred after weaning in the 1α(OH)ase^−/−^ mice with deficient 1,25(OH)_2_D production but intact Vdr, and treatment with exogenous 1,25(OH)_2_D_3_ normalized serum calcium.^(^ ^9^ ^)^ In contrast, in the Vdr^−/−^ mice with elevated endogenous levels of 1,25(OH)_2_D, deficiency of Vdr, resulted in hypocalcemia which was not corrected by exogenous 1,25(OH)_2_D_3_ treatment, indicating that both 1,25(OH)_2_D and the VDR are necessary for optimal intestinal absorption of calcium. Intestine‐specific deletion of the *Vdr* further demonstrated that when dietary calcium is low, the 1,25(OH)_2_D/VDR pathway is required to maintain normocalcemia and bone health by enhancing intestinal calcium absorption.^(^ ^13^ ^)^ 1,25(OH)_2_D increases intestinal calcium transport by regulating the expression of genes that transport Ca^2+^ by the transcellular, energy‐dependent pathway, including the apical Ca^++^ channel TRPV6, the intracellular Ca^++^ binding protein, Calbindin‐D9k and the Ca^++^‐ATPase, PMCA1b, which extrudes Ca^++^ at the basolateral membrane.^(^ ^14^ ^)^ Indeed, intestine‐specific transgenic expression of TRPV6 can recover calcium absorption and prevent rickets in Vdr^−/−^ mice.^(^ ^15^ ^)^ However, global gene deletion of TRPV6 has shown that although apical Ca^++^ uptake is the rate‐limiting step in 1,25(OH)_2_D‐mediated intestinal calcium absorption, additional Ca^++^ channels beside TRPV6 appear to be involved.^(^ ^16^ ^)^ Calcium absorption may also be facilitated by passive transport of calcium via 1,25(OH)_2_D‐regulated claudin‐2 and claudin‐12, major transmembrane components of tight junctions involved in paracellular calcium transport.^(^ ^17^ ^)^

## Calcitriol and Renal Phosphate Metabolism

Vdr^−/−^ mice have undetectable levels of FGF23,^(^ ^18^ ^)^ which is produced by osteoblastic/osteocytic cells, and 1,25(OH)_2_D can modulate renal phosphate re‐absorption by increasing FGF23, which suppresses expression of the renal cotransporters, Na/Pi‐2a and Na/Pi‐2c.^(^ ^19^ ^)^ Elevated FGF23 can in turn decrease Cyp27b1 and PTH expression, thus reducing 1,25(OH)_2_D levels and causing a rachitic/osteomalacic phenotype.^(^ ^20^, ^21^ ^)^ Dietary normalization of serum calcium and phosphate levels in VDR^−/−^ mice increases FGF23 levels, and the serum phosphate/calcium ratio appears to be a potent stimulator of FGF23.^(^ ^22^ ^)^ FGF23 null mice exhibited hyperphosphatemia, elevated 1,25(OH)_2_D levels, moderate hypercalcemia, and low PTH levels,^(^ ^23^ ^)^ and FGF23^−/−^/1α(OH)ase^−/−^ compound null mutant mice have low 1,25(OH)_2_D levels, increased PTH levels, decreased renal Na/Pi‐2a activity, hyperphosphaturia, and hypophosphatemia.^(^ ^24^ ^)^ The abnormal skeletal nodule formation and soft tissue calcifications found in FGF23^−/−^ mice disappeared in the FGF23^−/−^/1α(OH)ase^−/−^ mice, suggesting that at least some of the skeletal abnormalities found in FGF23^−/−^ mice are mediated through increased 1,25(OH)_2_D action.^(^ ^24^ ^)^ Osteoblast‐specific deletion of Cyp27b1 has indicated that production of 1,25(OH)_2_D in osteoblastic cells by extrarenal Cyp27b1, appears to be an intracrine stimulator of FGF23 production, which may be especially important in uremia.^(^ ^25^ ^)^ Crossing Cyp24 null mice with mice expressing elevated FGF23 increased 1,25(OH)_2_D levels and ameliorated their rachitic phenotype.^(^ ^26^ ^)^

## Calcitriol and Skeletal Homeostasis

### Effects on the growth plate

In both 1α(OH)ase^−/−^ mice and Vdr^−/−^ mice, typical histologic features of advanced rickets were observed, including widening of the epiphyseal growth plates, mainly because of a widened and disorganized hypertrophic zone, and inadequate mineralization of cartilage, of the primary spongiosa.^(^ ^3^, ^4^, ^8^, ^9^ ^)^ Although a rescue diet partly corrected these defects due to normalization of calcium,^(^ ^27^ ^)^ phosphate,^(^ ^28^ ^)^ and PTH, correction of the growth plate abnormalities, at least in some studies, required 1,25(OH)_2_D treatment for complete amelioration.^(^ ^9^ ^)^ The direct beneficial effects of 1,25(OH)_2_D in normalizing the growth plate has also been shown in other models of rickets.^(^ ^29^ ^)^ In view of the fact that osteoclast/chondroclast production at the chondro‐osseous junction may also be defective, diminished removal of hypertrophic chondrocytes by 1,25(OH)_2_D^(^ ^30^ ^)^ may occur in this region, leading to altered cartilage growth plate remodeling. Indeed, chondrocyte‐specific ablation of *Cyp27b1* led to an increase in the hypertrophic chondrocyte layer of embryonic bones, decreased expression of receptor activator of nuclear factor‐kappa B ligand (RANKL), a critical cytokine for osteoclast stimulation,^(^ ^31^ ^)^ and impaired osteoclastogenesis.^(^ ^30^ ^)^ Chondrocyte‐specific ablation of the Vdr also demonstrated delayed vascular invasion associated with a reduction in VEGF expression.^(^ ^32^ ^)^ These studies therefore confirmed a role for the Vdr and its ligand in the skeletal growth plate and endochondral bone formation.

### Effects on skeletal mineralization

An increase in osteoid, reflecting osteomalacia, was observed in both trabecular and cortical bone in both 1α(OH)ase^−/−^ mice and VDR^−/−^ mice. The effects of the 1,25(OH)_2_D/Vdr system to promote bone mineralization and eliminate osteomalacia appeared to be mainly indirect by enhancing intestinal calcium and phosphorus absorption and thus normalizing the serum mineral levels.

In intestine‐specific Vdr deletion, reduced calcium absorption was associated with normocalcemia, increased PTH, elevated 1,25(OH)_2_D, an increase in bone resorption, and impaired mineralization of bone.^(^ ^13^ ^)^ The impaired bone mineralization was associated with increased expression of the local mineralization inhibitors ectonucleotide pyrophosphatase phosphodiesterase (Enpp) 1, Enpp3, and progressive ankylosis (Ank), which are direct targets of 1,25(OH)_2_D and which regulate the relative osseous amounts of pyrophosphate to phosphate, thereby modulating mineralization. Levels of FGF23 were not, however, reported in those studies.

### Effects on bone turnover

In the presence of insufficient calcium absorption, 1,25(OH)_2_D levels may markedly increase and enhance bone turnover, leading to osteopenia. Furthermore, studies of osteoblast‐specific Vdr deletion, but not of osteocyte‐specific Vdr deletion, have shown that 1,25(OH)_2_D may act via the Vdr in osteoprogenitors and early osteoblasts to increase the ratio of RANKL relative to its inhibitor osteoprotegerin (OPG) and thus increase osteoclastogenesis and osteoclast activity. This results in increased osteoclastic bone resorption and calcium mobilization.^(^ ^13^ ^)^

In contrast, studies using 1α(OH)ase^−/−^ mice^(^ ^3^, ^9^ ^)^ or double homozygous knockout of 1α(OH)ase and the PTH gene (*Pth*)^(^ ^33^ ^)^ or double homozygous knockout of 1α(OH)ase and the calcium‐sensitive receptor gene (*Casr*),^(^ ^34^ ^)^ indicated that endogenous and exogenous 1,25(OH)_2_D can also directly stimulate osteoblastic bone formation via the Vdr, although the dose may be lower than that required for bone resorption.

### Calcitriol and teeth

Vdr^−/−^ mice have enamel and dentin abnormalities, and defective dental root resorption.^(^ ^35^, ^36^ ^)^ Normalizing serum calcium and phosphate levels in Vdr^−/−^ mice reversed resorption; however, enamel and dentin integrity was only partly restored. The dental volume, reparative dentin volume, and dentin sialoprotein immunopositive areas were also reduced in 1α(OH)ase^−/−^ mice,^(^ ^37^ ^)^ and in the mandibles of 1α(OH)ase^−/−^ mice, the cortical thickness, dental alveolar bone volume, and osteoblast number were also all decreased significantly, with accelerated alveolar bone loss.^(^ ^38^ ^)^ The SIRT1/FOXO3a signaling axis appears to play an important role in the prevention of mandibular bone loss by 1,25(OH)_2_D.^(^ ^39^ ^)^

### Effects on skeletal aging

**Fig 2:**
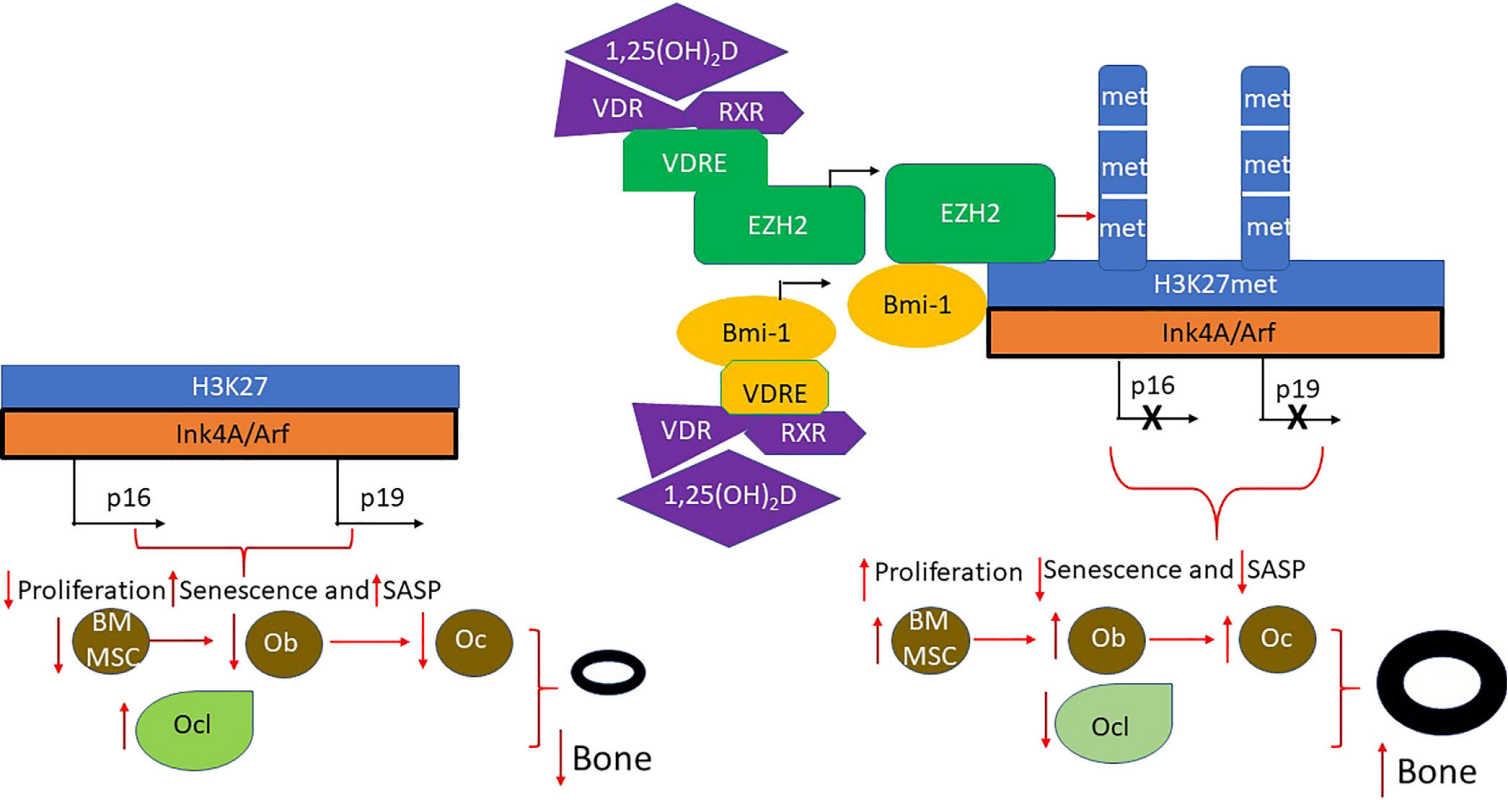
Model of regulation of bone mass, by 1,25(OH)_2_D, in aging mice with *cyp27b1* deficiency. In the absence of 1,25(OH)_2_D (left), p16 and p19 are transcribed off the Ink4A/Arf locus and induce decreased proliferation and increased senescence and SASP in cells of the osteoblast lineage, resulting in decreased bone formation, increased osteoclastic bone resorption, and decreased bone mass.^(^ ^44^ ^)^ Following treatment with 1,25(OH)_2_D (right), the hormone, bound to the VDR signals via VDREs to increase EZH2.^(^ ^44^ ^)^ and increase Bmi1.^(^ ^41^ ^)^ Bmi1 can directly associate with Ezh2,^(^ ^42^ ^)^ and Ezh2 trimethylates H3K on lysine residues at position 27. This inhibits p16 and p19 transcription and increases proliferation and reduces senescence and SASP.^(^ ^44^ ^)^ This results in increased osteoblastic bone formation, reduced osteoclastic bone resorption and increased bone mass.^(^ ^44^ ^)^

Bone mineral density, bone volume, Cyp27b1 renal protein expression, and circulating 1,25(OH)_2_D all decrease progressively with age in haploinsufficient 1α(OH)ase^+/−^ mice, and *Cyp27b1* gene haploinsufficiency accelerated age‐related bone loss and an osteoporotic phenotype, accompanied by declining osteoblastic bone formation and increasing osteoclastic bone resorption.^(^ ^40^ ^)^ The INK4a/ARF locus encodes the INK4 family of cyclin‐dependent kinase inhibitors, including p16^INK4a^, and a tumor suppressor p19/p14^ARF^.^(^ ^41^ ^)^ *Cyp27b1* gene haploinsufficiency was associated with increasing oxidative stress and DNA damage in bone, and with increased p16^INK4A^, capable of inducing cell cycle arrest in G1 phase, and p19^ARF^, which can induce both G1 and G2 arrest, thus inducing bone cell senescence and a senescence associated secretory phenotype (SASP). This appeared to then lead to inhibition of osteogenesis of mesenchymal stem cells and osteoblastic bone formation with increased osteoclastic bone resorption.^(^ ^41^ ^)^ Bmi1, a polycomb group (PcG) protein, appeared to play an important role in the prevention of 1,25(OH)_2_D deficiency‐induced bone loss, associated with reductions of p16/p19.^(^ ^41^ ^)^ Bmi1 repression of the Ink4A/Arf locus requires its direct association with and is dependent on the continued presence of Ezh2, the catalytic subunit of the Polycomb repressive complex 2 (PRC2), that can increase trimethylation of histone H3 at lysine 27 to produce the repressive mark H3K27me3 along the Ink4A/Arf locus, and thus repress p16 and p19 transcription.^(^ ^42^, ^43^ ^)^ In aging homozygous 1α(OH)ase^−/−^ mice in which serum calcium, phosphorus, and PTH levels were normalized by administering a rescue diet, age‐related bone loss was also increased by activation of p16/p19 signaling, and induction of senescence and SASP, reduction of osteoblastic bone formation, and accelerated osteoclastic bone resorption.^(^ ^44^ ^)^ Exogenous 1,25(OH)_2_D_3_ supplementation rescued the bone loss induced by 1,25(OH)_2_D deficiency by reversing these abnormalities. The VDR was shown to bind to a VDRE‐like sequence upstream of the histone methyltransferase Ezh2 gene promoter (suggested by bioinformatic analysis, confirmed by chromatin immunoprecipitation (ChIP)‐qPCR, and shown to be functional by luciferase assay), and Ezh2 was upregulated in 1,25(OH)_2_D_3_‐treated bone marrow mesenchymal stem cells (BM‐MSCs).^(^ ^44^ ^)^ When the status of H3K27me3 along the INK4a/ARF locus in BM‐MSCs was then examined, H3K27me3 was found to be enriched in the regions just upstream of the transcription start site (TSS) p16‐1, p19‐1, surrounding the TSS p16‐2, p19‐2, and within the intron immediately downstream of the TSS p16‐3, p19‐3 of p16^INK4a^/p19^ARF^ in 1,25(OH)_2_D_3_‐treated BM‐MSCs compared with vehicle‐treated BM‐MSCs, and the expression levels of p16^INK4a^ and p19^ARF^ were downregulated.^(^ ^44^ ^)^ Furthermore, the actions of 1,25(OH)_2_D_3_ were partially blocked following treatment with an Ezh2 inhibitor. The consequence was repression of p16/p19 transcription, suppression of oxidative stress and DNA damage, and reduction in bone cell senescence and SASP. This promoted the proliferation of, and the production of osteoblasts and osteocytes, and lead to stimulation of osteoblastic bone formation and inhibition of osteoclastic bone resorption (Fig. 2).

## Calcitriol and the Parathyroids

On a normal diet, when hypocalcemia is present in both 1α(OH)ase^−/−(^ ^3^, ^4^, ^9^ ^)^ and the Vdr^−/−(^ ^8^ ^)^ mice, increased circulating PTH concentrations and enlarged parathyroid glands occur. After dietary normalization of calcium, serum PTH concentrations fell in both 1α(OH)ase^−/−^, and VDR^−/−^mutants, indicating that raising the ambient calcium could alone normalize PTH secretion. However, in mice with parathyroid‐specific ablation of the VDR a mild increase in circulating PTH levels has been reported despite normocalcemia^(^ ^45^ ^)^; this was attributed to a decrease in expression of the parathyroid CaSR. In 1α(OH)ase^−/−^ mice on a calcium‐normalizing diet, parathyroid gland size was reduced, but remained moderately enlarged, and treatment with exogenous 1,25(OH)_2_D_3_ normalized parathyroid gland size.^(^ ^9^ ^)^ Consequently both calcium and 1,25(OH)_2_D may act cooperatively to diminish PTH production and parathyroid gland size. It is likely, however, that these interventions are most effective prior to the development of hyperparathyroidism.

## Selected Actions Beyond Mineral and Skeletal Homeostasis

### Calcitriol and skin

Cutaneous exposure to UVB irradiation is an important source of vitamin D by photolysis of its precursor, 7‐dehydrocholesterol (7DHC). 7DHC can be alternatively metabolized by a cholesterol side chain cleavage enzyme, cytochrome P450scc (CYP11A1), to 7‐dehydropregnenolone, which is then degraded by other steroidogenic enzymes.^(^ ^46^ ^)^ 25(OH)D per se is not, however, a substrate for CYP11A1. Skin‐specific androgen receptor deletion in male mice, has demonstrated that local androgen regulation of CYP11A1, may contribute to sex differences in UVB‐induced vitamin D production.^(^ ^47^ ^)^

Vdr^−/−^ mice develop alopecia, similar to humans with VDDR2A.^(^ ^8^ ^)^ This is in contrast to 1α(OH)ase^−/−^mice, or patients with VDDR1A.^(^ ^3^, ^4^ ^)^ Thus, studies in Vdr^−/−^ mice demonstrated that VDR actions independent of the 1,25(OH)_2_D ligand are required for cyclical regeneration of the hair follicle and for cutaneous homeostasis.^(^ ^48^ ^)^

Keratinocytes can also express Cyp27b1, generate 1,25(OH)_2_D locally,^(^ ^49^ ^)^ and respond to 1,25(OH)_2_D with altered proliferation and differentiation.^(^ ^50^ ^)^ 1,25(OH)_2_D signaling in keratinocytes acts with calcium, in part via CaSR and β‐catenin. β‐catenin acts as a transcription factor with VDR to enable proliferation but also acts, as a component of the membrane E‐cadherin–catenin complex, to facilitate differentiation.^(^ ^51^ ^)^ Deletion of VDR and/or CaSR in epidermal keratinocytes of mice disrupts epidermal differentiation, impairs wound healing,^(^ ^52^ ^)^ and predisposes to cancer.^(^ ^53^ ^)^ 1,25(OH)_2_D also promotes the innate immune function of keratinocytes by inducing the Toll‐like receptor 2 (TLR2) and its co‐receptor CD14. TLR2 and CD14 can then induce Cyp27b1, which increases 1,25(OH)_2_D production that then also induces the potent antimicrobial peptide, cathelicidin.^(^ ^54^ ^)^

### Calcitriol and skeletal muscle

VDR levels in skeletal muscle are normally low. Nevertheless, myocyte‐specific VDR deletion in mice reduced proportional lean mass, reduced voluntary wheel‐running distance, reduced average running speed, reduced grip strength, and increased proportional fat mass, although body size remained normal. Muscle expression of the cell cycle genes cyclin and cyclin‐dependent kinases, and of calcium‐handling genes and sarcoplasmic/endoplasmic reticulum genes were all decreased.^(^ ^55^ ^)^

### Calcitriol and cardiac muscle

Cardiomyocyte‐specific deletion of the VDR in mice produced a reduction in end‐diastolic and end‐systolic volume by echocardiography, and increased atrial natriuretic peptide and alpha skeletal actin gene expression. Furthermore, there was increased expression of modulatory calcineurin inhibitory protein 1 (MCIP1), a direct downstream target of calcineurin/nuclear factor of activated T cell (NFAT) signaling. Thus, 1,25(OH)_2_D/VDR signaling appears to exert antihypertrophic activity in the heart involving suppression of the prohypertrophic calcineurin/NFAT/MCIP1 pathway.^(^ ^56^ ^)^

### Calcitriol and adipose tissue

Fatty acids are a major energy source in the body, and white adipose tissue is a primary site where fatty acids are stored as triacylglycerols. Brown adipose tissue also stores and recruits fatty acids as a carbon source for uncoupled β‐oxidation during thermogenesis. Global Vdr^−/−^ mice exhibit a lean, white adipose tissue phenotype with increased levels of expression of the brown adipose tissue marker Ucp1 in the white adipose tissue. The 1,25(OH)_2_D/Vdr signaling appears to specifically modulate fatty acid composition in subcutaneous white adipose tissue by direct regulation of a specific elongase enzyme (Elovl3), which has an important role in brown fat biology.^(^ ^57^ ^)^ In contrast female mice with adipose‐specific Vdr deletion exhibited higher growth rates and increased visceral fat pad weight with elevated Ucp1 and Pparγ expression in white adipose tissue. Although the weight of the subcutaneous (mammary) fat pad was not increased in these mice on a high fat diet, mammary epithelial density and branching were significantly enhanced. Consequently 1,25(OH)_2_D/Vdr signaling in mature adipocytes may exert anti‐proliferative effects on the mammary epithelium.^(^ ^58^ ^)^

### Calcitriol and hypertension

In Vdr^−/−^ and 1αOHase^−/−^ mice, renin gene expression was increased causing increased renin conversion of angiotensinogen to angiotensin I and increased conversion of angiotensin I to angiotensin II via angiotensin converting enzyme (ACE). This lead to hypertension, cardiac hypertrophy, and increased water intake.^(^ ^59^, ^60^ ^)^ Angiotensin II, acting on AT1 receptors, exerts potent vasoconstrictor, pro‐fibrotic, and pro‐inflammatory effects. Conditional deletion of the Vdr from endothelial cells demonstrated increased blood pressure with angiotensin II infusion suggesting that the endothelial Vdr plays an important role in endothelial cell function and blood pressure regulation.^(^ ^61^ ^)^

Angiotensin‐(1–7) [Ang‐(1–7)], is a heptapeptide catalyzed by angiotensin converting enzyme 2 (ACE2) from Angiotensin II; it is protective against inflammation and oxidative damage through binding with Mas receptors (MasR), is a potent vasodilator, anti‐apoptotic, and anti‐proliferative agent and can counteract the harmful effects of the ACE/Ang II/AT1 pathway.^(^ ^62^ ^)^ Therefore, ACE2 is a negative regulator of ACE in the renin‐angiotensin system (RAS). ACE2 is a membrane protein found in nearly all body tissues with relatively higher expression in respiratory epithelial cells, type I and II alveolar cells, heart, blood vessels, kidney, and the gastrointestinal tract. In rats with diabetic kidney disease, calcitriol decreased ACE concentration, enhanced ACE2 concentration, and decreased ACE/ACE2 ratio.^(^ ^63^ ^)^

### Calcitriol and the immune system

The important role of vitamin D in immune system function was recognized early on in 1α(OH)ase^−/−^ mice and VDR^−/−^ mice.^(^ ^64^ ^)^ Vitamin D metabolic enzymes and the Vdr are present throughout the immune system, and Cyp27b1 production in immune cells such as macrophages and dendritic cells is induced by pathogen detection and regulated by a complex cytokine network including Interferon gamma (IFNγ), and CD14, a cofactor of toll‐like receptor 4 (TLR4), as well as by Pattern recognition receptor (PRR).^(^ ^65^ ^)^ Combined activation of the Janus kinase 3 (JAK‐STAT), p38 MAPK, and NF‐κB pathways occurs, with CCAAT/enhancer binding protein beta (C/EBPbeta) likely being the essential transcription factor controlling Cyp27b1 immune‐mediated transcription. Therefore, although immune cell Cyp27b1, in contrast to renal Cyp27b1, is not regulated by the factors controlling calcium and phosphate homeostasis, increased Cyp27b1 expression in macrophages can result in elevated circulating 1,25(OH)_2_D levels in granulomatous diseases such as sarcoidosis, which may lead to hypercalcemia.^(^ ^66^ ^)^

The 1,25(OH)_2_D generated locally in immune cells from circulating 25(OH)D regulates both innate and adaptive immunity.^(^ ^67^ ^)^ Thus, 1,25(OH)_2_D signaling increases expression of several types of proteins implicated in innate immune signaling, including PPRs, CD14/TLR4, as well as nucleotide‐binding oligomerization domain protein 2 (NOD2), and also stimulates expression of genes encoding antimicrobial peptides including cathelicidin, which has antiviral activity against enveloped viruses in vitro and influenza A.^(^ ^68^ ^)^ 1,25(OH)_2_D signaling through the VDR also increases the expression of several cytokines, including interleukin 1 (IL‐1), a core component of innate immune responses; the neutrophil chemokine IL‐8/CXCL8 is also induced, especially in macrophages infected with *Mycobacterium tuberculosis*.^(^ ^69^ ^)^

Signaling by 1,25(OH)_2_D also regulates the innate‐adaptive immune interface rendering dendritic cells less inflammatory. Thus signaling decreases peripheral inflammatory T cell (T helper cell type 1 [Th1] and Th17) responses and enhances production of Th2 and T‐regulatory (Treg) cells.^(^ ^70^ ^)^

The 1,25(OH)_2_D generated locally in immune cells also modulates adaptive immunity, generally suppressing inflammatory immune responses that underlie autoimmunity, eg, multiple sclerosis,^(^ ^71^ ^)^ and regulating allergic responses. For example, using both global 1α(OH)ase^−/−^ mice and mice with Cyp27b1 conditionally deleted from T‐cells, locally synthesized 1,25(OH)_2_D was found to participate in the control of IgE class‐switch recombination in B cells, suggesting that 1,25(OH)_2_D reduces type I sensitization.^(^ ^72^ ^)^

Both 1α(OH)ase^−/−(^ ^73^ ^)^ and Vdr^−/−^ mice^(^ ^74^, ^75^ ^)^ show increased severity of experimentally‐induced colitis that mimics inflammatory bowel disease, and treatment with 1,25(OH)_2_D_3_ improves experimental colitis in murine models. 1,25(OH)_2_D is important for regulating the immunity of gut mucosa via the modulation of innate immune barrier function, gut epithelial integrity, and the development and function of T cells. Thus, 1,25(OH)_2_D can prevent the onset of inflammatory bowel disease through the stabilization of microbiota homeostasis as well as ameliorate disease progression via anti‐inflammatory immune responses.^(^ ^76^ ^)^

1,25(OH)_2_D might be capable of reducing the severity of coronavirus disease 2019 (COVID‐19) infections through diverse mechanisms. These may include enhancement of cellular immunity by downregulating pro‐inflammatory cytokines and decreasing cytokine storm, and regulation of adaptive immunity by suppressing Th1 responses and promoting Treg cell induction. Nevertheless, 1,25(OH)_2_D may augment ACE2,^(^ ^77^ ^)^ the receptor for severe acute respiratory syndrome‐coronavirus‐2 (SARS‐CoV‐2), by which SARS‐CoV‐2, the cause of COVID‐19, enters the cell. However SARS‐CoV‐2 infection itself downregulates ACE2 activity, causing Ang II overaccumulation, which in turn may cause acute respiratory distress syndrome (ARDS) or fulminant myocarditis. Therefore, whether 1,25(OH)_2_D exerts a neutral, beneficial, or negative effect on SARS‐CoV‐2 infection remains to be determined.

### Calcitriol and malignancy

Some in vivo studies of vitamin D and malignancy in animals have crossed Vdr^−/−^ mice with models of tumors such as colon cancer generated in mice with mutated oncogenes (eg adenomatous polyposis coli [APC]).^(^ ^78^ ^)^ Others have used xenografted animal models to demonstrate tumor‐inhibitory effects of 1,25(OH)_2_D.^(^ ^79^ ^)^ In skin, spontaneous tumors were reported when both VDR and CaSR were deleted and the mice were placed on a low‐calcium diet.^(^ ^80^ ^)^ As well, in vitro studies of tumor cell lines and tumor cell cultures isolated from both humans and animals have shown inhibitory effects of 1,25(OH)_2_D or analogs. These studies have shown, for example, that 1,25(OH)_2_D_3_ inhibits the proliferation and promotes the differentiation of colorectal cancer cells through several mechanisms including: the induction of VDR binding to β‐catenin within the cell nucleus that prevent the formation of transcriptionally active TCF7L2/β‐catenin complexes; the upregulation of E‐cadherin at the plasma membrane where it attracts the newly synthesized β‐catenin protein; and the induction of the gene Dickkopf WNT signaling pathway inhibitor 1 (DKK1).^(^ ^81^, ^82^, ^83^ ^)^ An important antiproliferative mechanism of 1,25(OH)_2_D is the inhibition of the MYC gene, a major cell cycle regulator that may be induced by the WNT/β‐catenin pathway. 1,25(OH)_2_D directly represses MYC gene expression^(^ ^84^ ^)^ and indirectly reduces MYC activity by inhibiting the WNT/β‐catenin pathway, and inducing the antagonistic partner MAD/MXD1 of MYC.^(^ ^85^, ^86^ ^)^ 1,25(OH)_2_D also reduces cell proliferation by interfering with signaling by mitogens such as epidermal growth factor (EGF) and insulin‐like growth factor (IGF) 2 protein.^(^ ^87^ ^)^

No spontaneous tumors have, however, been reported in young mice lacking only 1,25(OH)_2_D or deficient in its receptor. In contrast, spontaneous tumors occurred in 11.5% of heterozygous 1α(OH)ase^+/−^ mice on a normal diet between 13 and 20 months of age, at which time serum 1,25(OH)_2_D levels were significantly reduced, and in 23.8% of homozygous 1α(OH)ase^−/−^ mice that survived over 12 months by being fed a diet to normalize calcium and phosphate.^(^ ^88^ ^)^ These 1,25(OH)_2_D‐deficient mice, greater than 1 year of age, developed diverse types of spontaneous tumors including breast cancer, squamous‐cell carcinoma of skin, soft tissue sarcoma, hepatocellular carcinoma, granulocytic sarcoma, endometrial stromal tumor, gastrointestinal stromal tumor, and pulmonary adenocarcinoma. Tumor development was associated with increased oxidative stress, cellular senescence, and production of SASP molecules, such as hepatocyte growth factor acting via its receptor c‐Met. Furthermore, treatment with 1,25(OH)_2_D_3_ prevented spontaneous tumor development.^(^ ^88^ ^)^

Specific mechanisms for 1,25(OH)_2_D actions have been postulated for different tumors. For example, in colon carcinomas, increased cell–cell adhesion, increased cell polarity, increased cell–extracellular matrix adhesion, and decreased epithelial‐mesenchymal transition; in breast cancer, inhibition of local estrogen synthesis and signaling that drives estrogen receptor‐positive (ER^+^) breast cancer; and in prostate cancer, interaction with androgen receptor (AR) signaling.^(^ ^89^ ^)^ Actions of 1,25(OH)_2_D on tumor‐associated stromal cells may include decreasing extracellular matrix remodeling, and decreasing promigratory effects on carcinoma cells, and in cancer stem cells, increasing cell–cell adhesion, increasing heterochromatization, and increasing organelles such as rough endoplasmic reticulum and Golgi, while decreasing tumorigenic cells.^(^ ^90^ ^)^ Additionally other anti‐tumor calcitriol actions may include enhancing DNA repair, and influencing activity of endothelial cells and cells of the immune system.^(^ ^91^ ^)^

## Conclusions

Using genetically modified mice, powerful tools have provided a large body of evidence supporting the role of 1,25(OH)_2_D in mineral and skeletal physiology. This data is generally translatable to humans and supports the critical importance of active vitamin D in mineral and skeletal homeostasis. However many important extraskeletal actions of 1,25(OH)_2_D have also been described in these mouse studies (Fig. 1). Although such diverse actions of active vitamin D have precedent, with pleiotropy exhibited by most other nuclear‐acting hormones (eg, glucocorticoids, thyroid hormones), these actions have often been more difficult to recapitulate in humans.^(^ ^92^ ^)^ Although the apparent discordance may reflect differences in mouse and human physiology, they may also reflect the fact that human clinical and epidemiologic studies often rely on administration of the prohormone, vitamin D, and/or on quantifying concentrations of the most abundant circulating, but generally inactive, metabolite, ie, 25(OH)D, to indirectly determine endocrine, paracrine, and intracrine actions of active 1,25(OH)_2_D. Future studies might consider the limitations of current methods of assessing vitamin D biology in humans in order to better assess the spectrum of actions of calcitriol, the active form of vitamin D.

### PEER REVIEW

The peer review history for this article is available at https://publons.com/publon/10.1002/jbm4.10434.
